# Increased awareness and knowledge of Lyme Borreliosis and tick bite prevention among the general population in France: 2016 and 2019 health barometer survey

**DOI:** 10.1186/s12889-021-11850-1

**Published:** 2021-10-08

**Authors:** Alexandra Septfons, Julie Figoni, Arnaud Gautier, Noémie Soullier, Henriette de Valk, Jean-Claude Desenclos

**Affiliations:** Santé publique France, Saint-Maurice, France

**Keywords:** Lyme Borreliosis, Tick bites, Epidemiology, Prevention, Knowledge

## Abstract

**Background:**

Lyme borreliosis (LB) is the most frequent tick-borne disease in France. In the absence of a vaccine, LB prevention mainly relies on reducing tick bites. In 2016, the French Ministry of Health launched a national plan against tick-borne infections, including a prevention component. To evaluate the impact of this prevention strategy, we assessed knowledge and practices of tick bite prevention using the 2016 and 2019 national surveys on health attitudes and beliefs known as the French Health Barometer.

**Methods:**

The Health Barometer is a repeated nationwide phone survey conducted annually on a random sample aged 18 to 75 years living in mainland France. In 2016 and 2019, participants were asked, among others, about their exposure to ticks, their behavior and practices regarding tick bites, and their knowledge about LB and its prevention.

**Results:**

In 2019, 30% of the population reported a lifetime tick bite and 6% in the last year, an increase from 25% and 4%, respectively, in 2016 (*p* < 0.001). In 2019, 25% of the population felt exposed to tick bites compared to 23% in 2016 (*p* < 0.001). The proportion of participants who had heard about LB and who considered themselves well informed respectively increased from 66% and 29% in 2016 to 79% and 41% in 2019, (*p* < 0.001). In 2019 compared to 2016, a greater part of the French population applied protective measures against tick bites, particularly wearing protective clothing (74% vs 66%, *p* < 0.001) and regular tick checks and prompt tick removal after exposure (54% vs 47%, *p* < 0.001).

**Conclusions:**

A substantial proportion of French residents are exposed to tick bites and apply protective measures. Our findings indicate a trend toward an increased knowledge and awareness of tick bites and LB between 2016 and 2019 in France. Our results can be used to target future information campaigns to specific age groups or at-risk areas in addition to the general population. However, we need to further study the barriers to the use of preventive measures.

## Background

Lyme borreliosis (LB), commonly referred to as Lyme disease by the general public, is the most common tick-borne infectious disease in France. It is caused by the bacteria *Borrelia burgdorferi* sensu lato and is transmitted to humans by a bite from an infected Ixodes tick [1].

The incidence of LB has increased over the last few decades in many European countries, including France in recent years [2–4]. In France, the incidence rate of GP consultations for LB increased from 51 per 100,000 inhabitants in 2015 to 104 per 100,000 inhabitants in 2018 (*p* < 0.001). LB is diagnosed in every region, with greater incidence rates in the eastern and central regions of France.

In the absence of a vaccine, preventive strategies mainly rely on the promotion of individual behaviors against tick bites. These include wearing protective clothing, applying tick repellent on the skin or clothing, checking for and removing ticks before or as soon as possible after they become attached after exposure in grassy and wooded environments, and staying on trails when in at-risk areas. In addition, the early detection of symptoms and consultation with a physician are essential, as early treatment can prevent the development of disseminated LB [5].

In September 2016, the French Ministry of Health issued a national plan to address LB and other tick-borne diseases [6]. One of the priorities of the plan was to “prevent tick-borne diseases.” Information campaigns were implemented or strengthened by regional and national health authorities and patient advocacy groups in order to inform the general public about the disease and the importance of effective preventive measures against tick bites and LB. These campaigns included broadcasting radio spots, distributing information leaflets, installing information boards at the entrance of woods and forests, and disseminating educational materials about the diagnosis, treatment, and prevention of the disease among healthcare professionals.

Nevertheless, information about the level of knowledge of the disease and the use of protective practices against tick bites among the general population is still needed in order to develop and adapt prevention and health promotion strategies. In several countries, studies have assessed the number of persons exposed to tick bites and the population’s general knowledge about LB and use of protective practices against tick bites [7–19]. However,relatively little is known about LB related knowledge and practices in France. A first study carried out in 2016 before the implementation of the national plan estimated that 4% of the population living in mainland France had been bitten by a tick in the last 12 months, 22% felt exposed to tick bites, and 28% reported feeling well informed about LB [20].

The aim of this study was to assess the knowledge, attitudes, and practices regarding LB and tick bite prevention based on the 2019 survey of the general population living in mainland France. By comparing the results of the 2016 and 2019 surveys, our analysis also aimed to compare the preventive behaviors and knowledge of LB before and after the launch of the national plan and thus contribute to evaluating its preventive component.

## Methods

### Survey and data collection

The Health Barometer Survey is a national cross-sectional telephone survey on health behaviors and perceptions that is regularly carried out in France. The survey includes questions on core health-related behavior and addresses additional health topics that vary from year to year. Questions on tick bite prevention and LB were included in the 2016 and 2019 surveys.

The Health Barometer sampling method is based on the random generation of landline and cellular phone numbers. The interviewee is randomly selected from the eligible household members via the landline or is the person answering the cellular phone. Each generated number is called up to 40 times at various times of the day and week in order to include individuals with limited availability. Only interviewees who are fluent in French are included. Estimates for the French population are obtained using weights that take into account the selection probability of the individual. They are then calibrated to adjust for the French population demographic structure in 2016 and 2018 as reported by the Labor Force Survey (conducted by the French National Institute for Statistics and Economic Studies, INSEE). The calibration parameters were gender by age in 10-year categories, region of residence, level of urbanization, size of household, and education level [21].

In 2016, 15,216 individuals aged between 15 and 75 years living in mainland France were included, with a participation rate estimated at 50%. In 2019, 10,352 individuals aged between 18 and 85 years living in mainland France were included, with a participation rate estimated at 51%. Among participants, 14,875 and 9611, respectively, were in the age group of interest for our study (18–75 years). The calculations showed that if the tick bite prevalence were 30%, it could be estimated with a precision of 0.0075 (sample size 14,875) and 0.01 (sample size 9611).

In addition to sociodemographic variables, the following data on LB and tick bites were collected for survey participants in 2016 and 2019: history of tick bites (lifetime and in the last 12 months); if the participant had consulted a healthcare professional; the perception of feeling exposed to tick bites (heavily exposed, exposed, not very exposed, not exposed); the use of protective measures against tick bites (often, sometimes, rarely, never) for at-risk exposures; the perception of their knowledge about LB, with questions about whether they had heard of it (yes, no), felt well informed (very well, quite well, not really, not at all), and were concerned about being infected (yes, somewhat yes, not really, not at all). In 2019, questions were added regarding the perception of potential health consequences of tick bites, the reason for having consulted a health professional, why people felt exposed to tick bites and where they were the last time that they were bitten [22, 23].

### Data analysis

For the statistical analyses and to aid the interpretation of the results, we dichotomized the perceptions of tick bite exposure (heavily exposed or exposed vs not really or not exposed), the use of protective measures (often or sometimes vs rarely or never), and the perception of knowledge about LB (very well or quite well informed vs not really or not at all). We also dichotomized the question about the perception of tick bite health consequences (always or sometimes vs never) and feelings of concern of being infected with LB (yes or somewhat yes vs not really or not at all).

We classified the administrative regions of residence into three categories according to the regional incidence of LB as estimated by the nationwide sentinel network of general practitioners (based on the average incidence of LB between 2011 and 2015 for the Health Barometer 2016 and between 2011 and 2018 for the Health Barometer 2019) [4]: Low incidence areas had less than 50 cases per 100,000 inhabitants per year, medium between 50 and 100 cases, and high more than 100 cases.

Variables about LB and tick bites were analyzed according to the sociodemographic characteristics available in the Health Barometer Survey: sex, age, education level (< secondary school, secondary school diploma, higher education degree), household monthly income (1st tercile [low], 2nd tercile [medium], 3rd tercile [high]), socio-professional category (farmers, craftsperson/tradesperson/business owner, executive/intellectual profession, intermediate profession, employees, blue-collar workers, no professional activity), level of urbanization of the place of residence (rural areas, less than 20,000 inhabitants, between 20,000 and 100,000 inhabitants, between 100,000 and 200,000 inhabitants, more than 200,000 inhabitants, Paris urban area), and region of residence.

For variables regarding tick bites, the perception of being exposed to tick bites, the use of protective measures against tick bites, the perception of information and knowledge about LB, we analyzed statistical differences according to sociodemographic characteristics. We also analyzed statistical differences between 2016 and 2019 for all explanatory variables assessed in both surveys.

We used the Chi-square tests to assess any statistical significance observed when comparing dummy variables. We considered *p*-values < 0.05 to be statistically significant.

We analyzed factors associated with the level of information about LB and the use of protective measures using multivariate logistic regression models. We used a backward selection approach to progressively eliminate variables with the highest *p*-value until only statistically significant variables remained (p-value< 0.05). Sociodemographic variables were systematically included in the model. The results were expressed as adjusted odds ratios (aOR). We considered *p*-values < 0.05 to be statistically significant.

### Ethical considerations

According to French law, this study did not have to obtain the approval of a national ethics committee, as it is not legally considered to be research involving human beings and relies only on the collection of anonymous data. Participants were adults who gave informed consent, and parental consent was obtained for 15–17-year-old interviewees in 2016 (not included in our analyses). No personal identifiers were recorded, and the anonymity of participants and the confidentiality of their data were guaranteed.

## Results

### Tick bites

In 2019, a high proportion of the French population (94%; 95% CI: 93–94) thought that a tick bite had health consequences (always for 29% (95% CI: 28–30) and often for 65% (95% CI: 64–66).

**Fig. 1:**
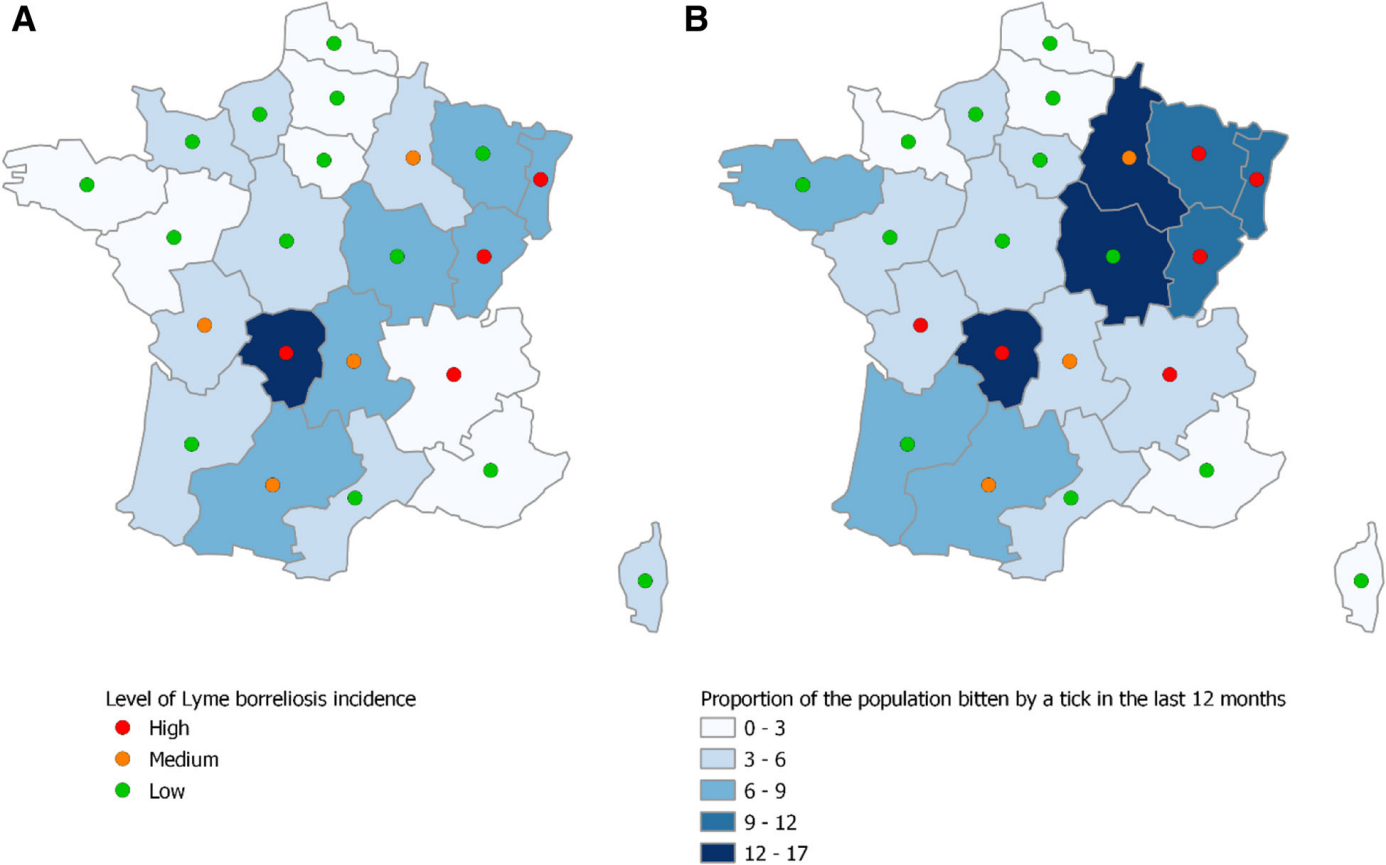
Proportion of the French population bitten by a tick in the last 12 months and the level of LB incidence by region according to Health Barometer 2016 (**A**) and Health Barometer 2019 (**B**), France

In 2019, 30% (95% CI: 29–32) of the French population declared that they had been bitten by a tick in their lifetime and 6% (95% CI: 5–7) during the past 12 months. As expected, the proportion of persons who experienced tick bites in their lifetime was significantly greater among those living in high and medium LB incidence areas compared to those living in low LB incidence areas, being respectively 40 and 38% versus 26% (*p* < 0.001). The same trends were also observed for those bitten by a tick in the past 12 months, with 8% in high and medium LB incidence areas compared to 5% in low LB incidence areas (*p* < 0.001). However, high LB incidence areas did not always coincide with a high frequency of tick bites (Fig. 1). The proportion of men who experienced tick bites in their lifetime was greater than that of women (32% vs 29%, *p* = 0.02). These proportions were not significantly different across age groups. The proportion of individuals with a history of tick bite(s) in their lifetime or during the past 12 months was greatest for those living in rural areas compared to urban areas (42% (95% CI: 40–45) and 9% (95% CI: 8–11), respectively), and for those working as farmers (46% (95% CI: 38–54) and 14% (95% CI: 10–21), respectively) or without a professional activity (32% (95% CI: 21–46) and 10% (95% CI: 4–23), respectively) compared to other socio-professional categories.

Among the bitten participants, the majority were in a forested area the last time that they were bitten (52%; 95% CI: 51–56). However, 22% (95% CI: 20–23) reported that they were in areas bordering fields and 16% (95% CI: 14–18) in a garden.

In terms of practices to remove the attached ticks, 67% (95% CI: 64–69) used tick removal devices or fine-tipped tweezers. Nevertheless, 18% (95% CI: 17–20) reported removing the tick with their hands, 6% (95% CI: 5–8) removed the ticks using other non-recommended methods (e.g., burning, ether, alcohol, knife), 6% (95% CI: 5–7%) waited for the tick to fall off by itself and 3% (95% CI: 2–4) did not remember.

Comparing the 2016 and 2019 results, we observed an increase in the proportion of people who had been bitten: 25% in 2016 versus 30% in 2019 reported a lifetime tick bite, while 4% in 2016 versus 6% in 2019 reported a tick bite during the last 12 months (*p* < 0.001) (Table 1). The proportion of those bitten during the last 12 months increased regardless of gender (Table 2). This increase was seen in all age groups, except for the 24–34 and 65–75 age groups (Table 2). In high and low LB incidence areas, the proportion of people bitten in the last 12 months increased between 2016 and 2019, from 5 to 8% and from 3 to 5%, respectively (*p* < 0.001) (Table 2). Nevertheless, the increased proportion of persons reporting tick bites was not significant in all regions (Fig. 1).

**Table 1:** Knowledge, behavior, and practices regarding tick bites and Lyme borreliosis in the French population by survey year, Health Barometer

	Health Barometer 2016		Health Barometer 2019		
	*N* = 14,875		*N* = 9611		chi2
**Have you ever been bitten by a tick?**	N	%	N	%	*p*-value
Yes	4056	25%	3123	30%	< 0.001
Less than 12 months ago	651	4%	620	6%	< 0.001
**If bitten, did you consult a physician?**					
Yes	126	20%	149	24%	NS
**If bitten, how did you remove the tick?**					
Tick removal devices or fine-tipped tweezers	NA	NA	2106	67%	
Other	NA	NA	1017	33%	
**Do you feel exposed to tick bites?**					
Yes (heavily exposed or exposed)	3774	23%	2580	25%	< 0.001
No (not really and not exposed)	11,101	77%	7031	75%	
**If feeling exposed, how often do you …**?					
Use skin repellent^a^	586	16%	458	18%	NS
Wear long sleeves and pants^a^	2502	66%	1919	73%	< 0.001
Check for and remove ticks^a^	1803	47%	1411	54%	< 0.001
At least one of the above three protective measures^a^	2918	76%	2177	83%	< 0.001
**Have you ever heard about Lyme disease?**					
Yes	10,746	66%	8176	79%	< 0.001
Never heard of it	4129	34%	1435	21%	
**Do you feel well informed about Lyme disease?**					
Yes	4986	29%	4401	41%	< 0.001
No	9889	71%	5210	59%	
If aware of Lyme disease:					
**What is the first symptom of Lyme disease?**					
Red skin rash	7179	66%	5679	69%	< 0.001
Other	3567	34%	2497	32%	

^a^Often or sometimes

**Table 2:** Proportion of the French population bitten by a tick in the last 12 months and who feel exposed to tick bites according to sociodemographic characteristics and survey year, Health Barometer 2016 and 2019, France

	Bitten by a tick in last 12 months			Feeling exposed to tick bites		
	2016 (*N* = 14,875)	2019 (*N* = 9611)	*p*-value	2016 (*N* = 14,875)	2019 (*N* = 9611)	*p*-value
	%	%		%	%	
Yes	4	6	< 0.001	23	25	< 0.001
**Sex**						
Male	4	7	< 0.001	22	24	0.03
Female	4	6	< 0.001	23	26	< 0.001
**Age** (years)						
18–24	4	7	< 0.001	13	16	NS
25–34	5	6	NS	20	24	0.008
35–44	4	6	0.02	22	28	0.001
45–54	3	5	0.006	25	25	NS
55–64	4	7	< 0.001	25	27	NS
65–75	5	5	NS	27	27	NS
**Level of LB regional incidence**						
High incidence	5	8	< 0.001	30	33	NS
Medium Incidence	6	8	NS	31	33	NS
Low incidence	3	5	< 0.001	20	21	0.02
**Level of urbanization of the place of residence**						
Rural	7	9	0.003	40	43	0.04
< 20,000 inhabitants	5	7	0.02	27	29	0.29
20,000–99,999 inhabitants	3	5	0.02	20	23	NS
100,000–199,999 inhabitants	3	5	NS	19	24	NS
> = 200,000 inhabitants	2	4	0.001	13	16	0.003
Paris urban area	2	4	0.04	12	11	NS
**Socio-professional category**						
Farmers	13	14	NS	64	63	NS
Craftsman/shopkeeper/business owner	3	6	0.04	25	28	NS
Executive/intellectual profession	4	7	< 0.001	23	21	NS
Intermediate profession	5	6	0.006	25	26	NS
Employees	3	5	0.002	20	23	0.01
Blue-collar workers	4	6	0.03	21	26	0.003
No professional activity	3	10	0.04	13	11	NS

Among those bitten by a tick, 20% (95% CI: 16–24) and 24% (95% CI: 20–28) consulted a physician because of the bite in 2016 and 2019, respectively.

### Feeling exposed to tick bites

In 2019, 25% (95% CI: 24–26) of the French population felt exposed to tick bites, increasing from 23% (95% CI: 22–24) in 2016 (*p* < 0.001) (Table 2).

Women felt more exposed than men (26% vs 24% in 2019, *p* < 0.001), while the feeling of being exposed increased with age (Table 2). Living in rural areas was associated with a feeling of being exposed to ticks: 43% or rural residents felt exposed compared to 10% of persons living in the Paris urban area (*p* < 0.001). Regarding socio-professional category, farmers felt the most exposed (63%; 95% CI: 54–71) (Table 2).

### Knowledge about Lyme borreliosis

In 2019, 79% (95% CI: 78–80) of the French population had heard about LB (Table 1) compared to only 66% (95% CI: 65–67) in 2016 (*p* < 0.001). In 2019, only 41% (95% CI: 40–42) felt well informed (Table 1), with this proportion being lower in 2016 (29%; 95% CI: 28–30) (Table 1). The proportion of individuals feeling well informed increased significantly regardless of gender, age, LB incidence area, level of urbanization, and socio-professional category (Table 3).

**Table 3:** Proportion of the French population who recognized *erythema migrans* as the first symptom of Lyme borreliosis and who felt well informed, according to sociodemographic characteristics and survey year for Health Barometer 2016 and 2019, France

	Knowing the first symptom of LB			Feeling well informed		
	2016 (*N* = 14,875)	2019 (*N* = 9611)	*p*-value	2016 (*N* = 14,875)	2019 (*N* = 9611)	*p*-value
	%	%		%	%	
**Yes**	66	68	< 0.001	29	41	< 0.001
**Sex**						
Male	64	65	NS	26	38	< 0.001
Female	67	71	< 0.001	32	44	< 0.001
**Age** (years)						
18–24	65	67	NS	16	26	< 0.001
25–34	66	67	NS	22	31	< 0.001
35–44	66	68	NS	25	35	< 0.001
45–54	66	69	NS	32	44	< 0.001
55–64	65	69	0.03	35	51	< 0.001
65–75	66	70	0.03	42	55	< 0.001
**Level of LB regional incidence**						
High incidence	72	75	NS	41	50	< 0.001
Medium incidence	66	68	NS	34	44	< 0.001
Low incidence	64	66	0.03	26	38	< 0.001
**Level of urbanization of the place of residence**						
Rural	68	71	NS	39	51	< 0.001
< 20,000 inhabitants	68	72	0.04	32	48	< 0.001
20,000–99,999 inhabitants	68	70	NS	27	42	< 0.001
100,000–199,999 inhabitants	62	68	NS	27	45	< 0.001
> = 200,000 inhabitants	63	65	NS	25	35	< 0.001
Paris urban area	61	64	NS	21	30	< 0.001
**Socio-professional category**						
Farmers	70	74	NS	45	54	NS
Craftsman/shopkeeper/business owner	61	68	0.03	30	41	< 0.001
Executive/intellectual profession	66	71	0.005	34	50	< 0.001
Intermediate profession	68	70	NS	34	45	< 0.001
Employees	65	69	NS	29	40	< 0.001
Blue-collar workers	64	63	NS	22	33	< 0.001
No professional activity	58	70	NS	16	33	0.0142

Female gender (aOR = 1.1; 95% CI: 1.1–1.2) and age over 45 years (aOR45–54 = 1.9; 95% CI: 1.5–2.4; aOR55–64 = 2.4; 95% CI: 1.9–3.1; and aOR65–75 = 3.0; 95% CI: 2.4–3.9) were associated with feeling better informed about LB (Table 4). Higher levels of education (aOR = 1.2; 95% CI: 1.0–1.4) and medium (aOR = 1.2; 95% CI: 1.0–1.4) or high (aOR = 1.3; 95% CI: 1.1–1.5) household monthly income were also associated with feeling better informed about the disease. People who lived in rural areas or in cities of less than 200,000 inhabitants were more likely to feel better informed than those living in the Paris urban area (Table 4).

**Table 4:** Factors associated with feeling well informed about Lyme borreliosis, Health Barometer 2019

	Feeling well informed			
	*N* = 9611			
	%	aOR	95% CI	
**Sex**				
Male	38%	ref		
Female	44%	1.1***	1.1	1.2
**Age** (years)				
18–24	26%	ref		
25–34	31%	1.1	0.8	1.4
35–44	35%	1.2	1.0	1.6
45–54	44%	1.9***	1.5	2.4
55–64	50%	2.4***	1.9	3.1
65–75	55%	3.0***	2.4	3.9
**Level of LB regional incidence**				
Low incidence	38%	ref		
Medium incidence	44%	1.1	0.9	1.3
High incidence	50%	1.3***	1.1	1.5
**Education level**				
< Secondary school level	39%	ref		
Secondary school diploma	39%	1.1	0.9	1.3
Higher education degree	46%	1.2*	1.0	1.4
**Household monthly income**				
1st tercile (low)	34%	ref		
2nd tercile	44%	1.2*	1.0	1.4
3rd tercile (high)	48%	1.3***	1.1	1.5
Refusal to answer	37%	1.0	0.8	1.3
**Level of urbanization of the place of residence**				
Paris urban area	30%	ref		
Rural	51%	1.5***	1.3	1.8
< 20,000 inhabitants	48%	1.5***	1.3	1.9
20,000–99,999 inhabitants	42%	1.3*	1.0	1.6
100,000–199,999 inhabitants	45%	1.4**	1.1	1.9
> = 200,000 inhabitants	35%	1.1	0.9	1.3
**Socio-professional category**				
Intermediate profession	45%	ref		
Farmers	54%	1.0	0.6	1.5
Craftsman/shopkeeper/business owner	41%	0.9	0.7	1.2
Executive/intellectual profession	50%	1.3***	1.1	1.6
Employees	40%	0.9	0.7	1.0
Blue-collar workers	33%	0.8*	0.7	1.0
No professional activity	33%	1.2	0.6	2.4
**Have you ever been bitten by a tick?**				
No	37%	ref		
Yes	53%	1.5***	1.4	1.7
**Feeling exposed to tick bites**				
No	38%	ref		
Yes	53%	1.3***	1.1	1.4
**Being concerned about having LB**				
No	33%	ref		
Yes	58%	2.0***	1.8	2.3
**Thinking tick bites can have health consequences**				
No	7%	ref		
Yes	44%	5.5***	3.7	8.2

*** *p* < 0.001; ** *p* < 0.01; * *p* < 0.05. aOR: adjusted odds ratio. 95% CI: 95% Confidence interval

Lifetime tick bite (aOR = 1.5; 95% CI: 1.4–1.7), feeling exposed to tick bites (aOR = 1.3; 95% CI: 1.1–1.4), being concerned about having LB (aOR = 2.0; 95% CI: 1.8–2.3), and thinking that tick bites have health consequences (aOR = 5.5; 95% CI: 3.7–8.2) were all strongly associated with a feeling of being well informed about LB (Table 4).

To assess the level of knowledge about LB, we asked respondents who had heard about LB to describe the first symptoms in the natural course of LB. In 2019, 69% (95% CI: 67–70) of the French population mentioned a “red skin rash” as the first symptom of LB, slightly more than in 2016 (66%; 95% CI: 65–67) (Tables 1 and 3). This proportion increased mostly for women, those aged over 55 years, and those living in a low incidence area for LB (Table 3).

We also found that being a woman (aOR = 1.3; 95% CI: 1.2–1.5) and being aged over 35 years (aOR35–44 = 1.8; 95% CI: 1.4–2.4; aOR45–54 = 1.5; 95% CI: 1.2–2.0; aOR55–64 = 1.5; 95% CI: 1.2–2.0; aOR65–75 = 1.6; 95% CI: 1.2–2.1) were associated with a greater feeling of concern about being infected with LB. On the contrary, having had one or more lifetime tick bites and living in a low incidence area for LB were associated with a lower level of concern about being infected with LB (respectively, aOR = 0.7; 95% CI: 0.6–0.8 and aOR = 0.7; 95% CI: 0.6–0.8).

### Protective measures among persons feeling exposed to tick bites

In 2019, among those feeling exposed to tick bites, the two most common protective measures used against tick bites were wearing protective clothing and checking the body for ticks after outdoor activities. About 73% reported using protective clothing (57% often), while 54% reported performing body checks (34% often) (Table 1). A much smaller proportion used tick repellent (18%). Despite feeling exposed to tick bites, a large proportion of the population declared never checking their skin for ticks (34%) or using protective clothing (18%). However, 83% reported using at least one of these three protective measures, while 45% reported both using protective clothing and performing body checks (Table 5).

**Table 5:** Proportion of the French population using protective measures against tick bites according to sociodemographic characteristics and survey year, Health Barometer 2016 and 2019, France

	Wearing long sleeves and pants			Checking for and removing ticks			Using at least one of the three protective measures^**a**^		
	2016 (*N* = 14,875)	2019 (*N* = 9611)	*p*-value	2016 (*N* = 14,875)	2019 (*N* = 9611)	*p*-value	2016 (*N* = 14,875)	2019 (*N* = 9611)	*p*-value
	%	%		%	%		%	%	
**Yes (often/sometimes)**	66	74	< 0.001	47	54	< 0.001	76	83	< 0.001
**Sex**									
Male	67	72	0.02	44	51	0.006	77	82	0.006
Female	64	74	< 0.001	50	57	0.002	76	84	< 0.001
**Age** (years)									
18–24	56	58	NS	51	46	NS	73	71	NS
25–34	65	72	NS	56	56	NS	75	81	NS
35–44	65	72	0.03	52	58	NS	78	84	NS
45–54	67	69	0.3	45	54	0.01	76	81	NS
55–64	68	79	< 0.001	43	54	< 0.001	79	88	< 0.001
65–75	66	80	< 0.001	41	49	0.01	75	88	< 0.001
**Level of LB regional incidence**									
High incidence	67	76	< 0.001	55	64	0.01	81	87	0.006
Medium incidence	68	68	NS	55	60	NS	79	82	NS
Low incidence	64	73	NS	43	47	0.03	74	81	< 0.001
**Level of urbanization of the place of residence**									
Rural	68	75	< 0.001	50	58	< 0.001	79	85	< 0.001
< 20,000 inhabitants	67	72	NS	46	54	0.02	76	82	NS
20,000–99,999 inhabitants	68	76	NS	50	56	NS	79	87	0.03
100,000–199,999 inhabitants	58	82	< 0.001	50	64	NS	71	89	0.001
> = 200,000 inhabitants	59	70	0.01	42	44	NS	71	78	NS
Paris urban area	65	67	NS	43	41	NS	74	78	NS
**Socio-professional category**									
Farmers	70	68	NS	47	57	NS	82	79	NS
Craftsman/shopkeeper/business owner	55	71	0.004	34	53	< 0.001	68	81	0.01
Executive/intellectual profession	68	72	NS	48	53	NS	76	79	NS
Intermediate profession	66	73	0.005	53	51	NS	78	83	0.03
Employees	63	76	< 0.001	46	57	< 0.001	74	87	< 0.001
Blue-collar workers	70	74	NS	46	54	0.04	80	83	NS
No professional activity	56	57	NS	44	30	NS	66	76	NS

^a^Using repellent, wearing long sleeves and pants, or checking for and removing ticks

Older age and being female were positively associated with the use of protective measures (Table 6). Compared to young adults (18–24 years), those aged 55 years and older were more likely to wear long sleeves and pants or use at least one of the three recommended protective measures (Table 6). Women were more likely than men to use skin repellent (aOR 1.4; 95% CI: 1.2–1.6) and more likely to wear long sleeves and pants and check for and remove ticks after exposure (aOR 1.1; 95% CI: 1.0–1.3) (Table 6).

**Table 6:** Factors associated with the use of protective measures for people who felt exposed to tick bites, Health Barometer 2019

	Using skin repellent				Wearing long sleeves and long pants				Checking for and removing ticks				At least one of the three protective measures^**a**^					Wearing long sleeves and pants AND checking for and removing ticks		
	*N* = 2580				*N* = 2567				*N* = 2555				*N* = 2571					*N* = 2572		
	%	aOR	95% CI		%	aOR	95% CI		%	aOR	95% CI		%	aOR	95% CI		%	aOR	95% CI	
**Sex**																				
Male	13%	ref			72%	ref			51%	ref			82%	ref			41%	ref		
Female	22%	1.4***	1.2	1.6	74%	1.0	0.9	1.1	57%	1.1	1.0	1.2	84%	0.9	0.7	1.2	48%	1.1*	1.0	1.3
**Age** (years)																				
18–24	13%	ref			58%	ref			46%	ref			71%	ref			34%	ref		
25–34	16%	1.2	0.6	2.6	72%	1.6	1.0	2.7	56%	1.0	0.6	1.8	81%	1.4	0.8	2.5	47%	1.2	0.7	2.1
35–44	18%	1.4	0.7	2.9	72%	1.7*	1.0	2.9	58%	1.0	0.6	1.7	84%	1.7	0.9	2.9	48%	1.2	0.7	2.0
45–54	18%	1.3	0.7	2.7	69%	1.4	0.8	2.2	54%	0.8	0.5	1.3	81%	1.2	0.7	2.2	43%	0.9	0.5	1.5
55–64	19%	1.5	0.7	2.9	79%	2.2***	1.3	3.7	54%	0.8	0.4	1.3	87%	2.0*	1.1	3.4	48%	1.0	0.6	1.8
65–75	18%	1.4	0.7	2.8	80%	2.2***	1.3	3.8	49%	0.6	0.4	1.0	88%	2.0*	1.1	3.6	41%	0.8	0.4	1.3
**Level of LB regional incidence**																				
Low incidence	20%	ref			73%	ref			47%	ref			81%	ref			40%	ref		
Medium incidence	15%	0.8	0.6	1.3	68%	0.7*	0.5	1.0	61%	1.4	1.0	2.0	82%	0.9	0.6	1.3	46%	1.0	0.7	1.4
High incidence	17%	1.1	0.8	1.5	76%	0.9	0.7	1.2	64%	1.5***	1.2	2.0	87%	1.2	0.8	1.6	53%	1.3*	1.0	1.6
**Education level**																				
< Secondary school level	20%	ref			77%	ref			53%	ref			85%	ref			46%	ref		
Secondary school diploma	16%	0.8	0.6	1.2	69%	0.7	0.5	1.0	49%	0.7*	0.5	1.0	80%	0.7	0.5	1.0	39%	0.7	0.5	0.9
Higher education qualification	15%	0.8	0.5	1.1	71%	0.8	0.6	1.1	58%	1.2	0.9	1.6	83%	0.9	0.6	1.4	46%	1.0	0.7	1.3
**Household monthly income**																				
1st tercile (low)	21%	ref			75%	ref			55%	ref			85%	ref			46%	ref		
2nd tercile	17%	0.7	0.5	1.0	72%	0.8	0.6	1.1	55%	1.0	0.7	1.3	81%	0.7	0.5	1.0	47%	1.0	0.8	1.4
3rd tercile (high)	14%	0.6*	0.4	0.9	73%	0.9	0.6	1.2	52%	0.9	0.6	1.2	84%	0.9	0.6	1.4	41%	0.8	0.6	1.2
Refusal to answer	18%	0.8	0.5	1.4	74%	1.1	0.7	1.7	47%	0.8	0.5	1.2	83%	1.0	0.6	1.7	40%	0.9	0.6	1.4
**Level of urbanization of the place of residence**																				
Paris urban area	23%	ref			67%	ref			41%	ref			77%	ref			35%	ref		
Rural	16%	0.5*	0.3	0.9	75%	1.2	0.8	2.0	59%	1.3	0.8	2.0	85%	1.2	0.7	2.2	48%	1.2	0.7	1.8
< 20,000 inhabitants	19%	0.7	0.4	1.2	72%	1.1	0.7	1.8	54%	1.2	0.7	1.9	87%	1.0	0.5	1.7	45%	1.1	0.7	1.8
20,000–99,999 inhabitants	22%	0.8	0.4	1.5	76%	1.4	0.8	2.4	56%	1.5	0.9	2.5	90%	1.5	0.8	2.9	47%	1.4	0.8	2.4
100,000–199,999 inhabitants	24%	0.9	0.4	2.1	82%	2.2*	1.1	4.5	64%	2.1*	1.1	4.1	78%	2.4	1.0	6.1	56%	2.1*	1.1	4.1
> = 200,000 inhabitants	14%	0.5*	0.3	0.9	70%	1.1	0.7	1.9	44%	1.0	0.6	1.7	77%	1.0	0.6	1.8	36%	1.0	0.6	1.7
**Socio-professional category**																				
Intermediate profession	16%	ref			73%	ref			50%	ref			83%	ref			41%	ref		
Farmers	11%	0.7	0.3	1.7	68%	0.7	0.4	1.1	57%	1.4	0.8	2.4	79%	0.6	0.3	1.1	45%	1.3	0.8	2.1
Craftsman/shopkeeper/business owner	15%	0.9	0.5	1.5	71%	0.8	0.5	1.2	53%	1.2	0.8	1.8	81%	0.8	0.5	1.3	43%	1.1	0.7	1.7
Executive/intellectual profession	15%	1.1	0.7	1.6	72%	0.9	0.6	1.3	53%	1.0	0.8	1.4	79%	0.7	0.4	1.0	45%	1.2	0.9	1.6
Employees	22%	1.1	0.8	1.7	76%	1.1	0.8	1.5	57%	1.5**	1.1	2.1	87%	1.4	1.0	2.1	47%	1.4*	1.0	1.8
Blue-collar workers	18%	1.2	0.8	1.9	74%	1.0	0.7	1.4	54%	1.4	0.9	2.0	83%	1.0	0.6	1.5	47%	1.4	1.0	2.0
No professional activity	29%	3.5	0.4	32.5	57%	0.7	0.1	3.4	29%	0.5	0.1	3.1	76%	1.0	0.1	6.6	10%	0.2	0.0	1.5
**Have you ever been bitten by a tick?**																				
No	16%	ref			70%	ref			41%	ref			78%	ref			33%	ref		
Yes	20%	1.4**	1.1	1.9	79%	1.6***	1.3	2.0	72%	3.7***	3.0	4.7	90%	2.5***	1.9	3.4	61%	3.3***	2.6	4.0
**Feeling concerned about having LB**																				
No	11%	ref			68%	ref			40%	ref			77%	ref			32%	ref		
Yes	23%	2.2***	1.6	3.0	78%	1.5***	1.2	1.9	64%	2.5***	2.0	3.1	88%	2.0***	1.5	2.7	54%	2.3***	1.8	2.8
**Thinking tick bites can have health consequences**																				
No	36%				49%	ref			41%	ref			70%	ref			32%	ref		
Yes	17%				74%	2.8*	1.0	7.7	54%				84%				45%			
**Feeling well informed about LB**																				
No	17%				68%	ref			46%	ref			78%	ref			37%	ref		
Yes	18%				78%	1.4**	1.1	1.8	61%	1.7***	1.3	2.1	88%	1.7***	1.3	2.3	51%	1.6***	1.3	1.9

*** *p* < 0.001; ** *p* < 0.01; * *p* < 0.05. aOR: adjusted odds ratio. 95% CI: 95% confidence interval, ^**a**^Using repellent, wearing long sleeves and pants, or checking for and removing ticks

Participants who reported experiencing one or more tick bites in their lifetime were more likely than those who had not (aOR 3.7; 95% CI: 3.0–4.7) to declare checking their body for ticks after being outdoors. They were also more likely to wear long sleeves and pants (aOR 1.6; 95% CI: 1.3–2.0) (Table 6).

In high LB incidence areas, people were more likely to declare checking for and removing ticks than in low incidence areas (aOR 1.5; 95% CI: 1.2–2.0) (Table 6).

Feeling well informed was positively associated with the use of protective measures. Thus, people were more likely to declare using protective measures if they felt well informed about LB (Table 6). Feeling concerned about being infected with LB was also positively associated with the use of protective measures (Table 6).

The proportion of persons applying protective measures against ticks, wearing protectives clothing, and checking for and removing ticks after exposure increased significantly between 2016 and 2019 (Table 1). This increase was observed for both men and women, particularly in the oldest age groups, high LB incidence areas, and rural areas (Table 5).

## Discussion

In this national study, we assessed preventive behaviors and knowledge of LB at the population level in mainland France in 2016 and 2019. Our results showed the progress of preventive behaviors and knowledge between 2016 and 2019, which may suggest a positive impact of the national LB plan. These findings also allow us to better target information campaigns to strengthen knowledge and practices regarding LB and tick bite prevention in France for specific population groups and territories. These results are crucial for developing or adapting programs for the prevention of tick-borne diseases.

We observed that around one-third of the population had been bitten by a tick in their lifetime, although a higher proportion of people felt exposed to tick bites and were concerned about LB. In rural and high LB incidence areas, the proportion of those reporting tick bites was even higher.

The proportion of persons bitten by ticks in their lifetime was greater in high LB incidence areas, but it did not completely correlate. For some regions, we observed a high incidence of tick bites despite the relatively low incidence of LB.

In France, the vector of LB, *Ixodes ricinus*, occurs nationwide, except around the Mediterranean basin as the climate is too dry [24]. Several areas in France can be considered at risk of LB transmission based on the presence of infected ticks, competent reservoir hosts, and favorable climatic and geographic characteristics (high humidity, moderate to heavy rain, adequate vegetation with grasslands, forests, or urban gardens and parks). However, the ecology of LB depends on multiple interactions, especially between humans, the vector – the pathogenic agent (*B. burgdorferi s.l*) –, and vertebrate reservoir hosts. At present, no nationwide data are available on the prevalence of *Borrelia* infection in ticks in France. However, a few regional studies have explored the rate of *Borrelia* infection in ticks in the main high-risk areas [5, 24].

Nevertheless, we highlight that the majority (52%) of tick bites occurred in a forest area, while 22% occurred in areas bordering fields and 16% in gardens. Our study shows a greater proportion of tick bites among outdoor workers like farmers. Outdoor workers are known to be at risk of LB as an occupational hazard, not to mention their recreational activities and their often-rural place of residence [5]. Data in the literature are scarce on the proportion of LB cases attributed to occupational exposure. High seroprevalence rates have been reported in forest rangers in France: 14.1% in northeast France [25] and 15.2% in the Île-de-France region [26].

In this context, it is important to reinforce information campaigns in high-risk areas, especially zones with a high incidence of tick bites and LB as well as rural and forest areas. However, since a significant proportion of the French population travel around the country every year or can be exposed to tick bites through recreational activities in the countryside, forests, urban parks, or private gardens with dense vegetation, information about LB and tick bite prevention should also target those living outside high-risk or forest areas.

We observed a slight increase in the proportion of people who reported a tick bite in 2019 compared to 2016 (6% vs 4%). We also found an increase in LB incidence over the last decade [4].

Since information about ticks and tick bites also increased during this period, the declaration of tick bites might have increased partly because of a better recognition of ticks. To fully understand the increase in LB incidence, we need further information about the distribution and density of infected ticks and reservoir hosts, as well as the interactions between humans, vectors, and reservoirs.

The proportion of persons who use preventive measures ranged from 18% for tick repellents to 54% for checking for ticks on the body and 73% for protective clothing. This suggests that many people are informed about the risk and accept using these measures. However, we also noted that these measures are not systematically applied, as only 34% of participants declared often performing body checks and 57% often using protective clothing.

The use of protective measures (protective clothing and checking for and removing ticks after exposure) was positively associated with being a woman, living in a populated city, being an employee, being exposed to ticks, being concerned about having LB, and feeling well informed about the disease. We also found that wearing long trousers and long-sleeved clothing was more common among those over 50 years. These findings are in accordance with studies in other countries, which found similar results on the use of preventive measures (lower for tick repellents and higher for protective clothing), the association with demographic factors such as older age groups and women, and the fact that being concerned and feeling well informed about LB are predictive of protective behavior [8, 9, 11, 13, 16, 18].

In addition, several factors such as being male, being under 45 years, living in highly urban areas, not feeling exposed to tick bites, and living in a low LB incidence area were associated with feeling not well informed about LB. We stress that information about tick bite prevention should target the entire French population, including medium- and low-risk areas, since a large majority of people can be exposed to tick bites and should be aware about the protective measures. However, our results indicate that more efforts should be made to reach men and younger people by adapting the information campaigns and media.

Despite the existence of efficient risk-reducing measures such as protective clothing that prevents ticks from attaching, effective repellents against tick bites, and the removal of ticks within 24 to 48 h to reduce the risk of LB transmission, [5, 27–29], the use of these measures is imbalanced and can be surprisingly low, especially in terms of checking the body and using repellent. Other studies have similarly shown that the use of repellent is the least commonly reported protective practice [8, 9, 11, 16, 18], probably due to the high cost and the perception that these products may be toxic to the skin, especially in the case of high or daily exposure.

We also noted that 67% of people who had been bitten by a tick used a tick remover as recommended [5]. However, 24% applied methods that are not recommended such as using oil, ether, or other products to facilitate tick removal. No study has demonstrated the effectiveness of such products [5] and the use of tick removers should continue to be promoted as a priority preventive measure against LB transmission.

Only 69% of the population who had heard about LB knew that *erythema migrans* was the first symptom in the natural course of LB. Because the early detection of symptoms and consultation with a physician is essential to prevent the development of disseminated LB, communication efforts should be strengthened regarding the knowledge and recognition of *erythema migrans*.

To optimize interventions to increase the frequency of preventive measures, a better understanding of the barriers to their use is necessary (lack of knowledge, discomfort, costs, etc.). In the Netherlands, the main barrier to checking for ticks after exposure was the low perception of risk and the fact that many persons could not recognize ticks [8]. Other studies suggested that the low proportion of repellent use could be due to the uncertainty of its efficacy as described in the Netherlands, the fear of toxic effects, or the lack of knowledge [8] . The cost of these products could also be an additional barrier. As also suggested in the Netherlands, the low proportion of people wearing protective clothing or checking for ticks could be explained by the discomfort (wearing protective clothing in summer is too hot), image issues (looking ridiculous with trousers tucked into socks), and lack of information about or access to tick removal devices [16]. Overall, risk perception and level of knowledge play an important role in the implementation of protective measures [16]. In our study, we show that being concerned about having LB and feeling well informed are positively associated with the use of protective behavior. The challenge is implementing balanced messages and campaigns to actively inform the population about the risks associated with tick bites and the proven effectiveness of the available measures in order to encourage reasonable precaution without provoking fear [30, 31].

In 2019 compared to 2016, a greater proportion of the population applied protective measures against tick bites and tick-borne diseases (wearing protective clothing, carrying out regular tick checks, promptly removing ticks after exposure). Furthermore, the proportion of those who had heard about LB and considered themselves well informed also increased over the last 3 years. These increases were greater in the oldest age groups, high LB incidence areas, and rural areas.

The fact that better knowledge is associated with better protection suggests a positive impact of the information campaigns. A higher level of knowledge is thought to positively influence protective behavior [8]. Since the launch of the national plan against tick-borne diseases, many information campaigns have been implemented or strengthened by regional or national health authorities and patient advocacy groups to inform the general public about the disease and preventive measures against tick bites. These information campaigns may have had a positive effect by raising awareness and increasing the level of information. Our study also showed some heterogeneity in the perceptions about LB information. The challenge will now be to take these results into account and apply them to prevention programs that reach the entire French population. As men and younger age groups (especially children and young adults) are less knowledgeable about LB, they should particularly be targeted with information about the disease and protective practices.

LB is also an increasing societal issue in France and the subject of public debates and sometimes controversies, particularly with regard to persisting post-treatment symptoms and the existence of a chronic version of LB. As a result, media coverage of LB has increased over the last few years, with many articles highlighting the different points of view of the scientific community, health authorities, health professionals, and patient advocacy groups [32]. This increased media coverage might also have had a positive impact on the general population’s level of awareness about ticks and tick bites and their knowledge about preventive measures. However, this campaign may also have contributed to the dissemination of rumors and beliefs with limited scientific evidence and increased the perceived seriousness of the disease by increasing fear. Thus, it is possible that in such a context, risk perceptions about LB could be socially amplified through the media. It is therefore important to ensure that scientifically sound and valid information is available and that information and social media campaigns are based on scientific evidence and adequately communicated to the general public.

We should acknowledge several limitations of the present study. A first limitation is the potential selection bias given the selective non-responses to our survey. To mitigate this potential source of bias, we weighted our analyses, thus taking into account the selection probability and adjusting for the sociodemographic structure of the French population. Additionally, refusals to participate in the survey were probably not related to its topic, as the survey presentation given to respondents did not mention LB. Second, our study shares the usual limitations of surveys based on self-reporting [33]. As a consequence, with the use of closed questions, participants’ knowledge of LB symptoms might have been overestimated. Likewise, the proportion of those reporting preventive measures against tick bites should be interpreted with caution, as we considered that protective measures were applied when survey participants used them sometimes or often. Furthermore, because these questions about preventive measures were only asked for the population who felt exposed to tick bites, we have reason to believe that the proportion of people using protective measures might be even lower in the general population. Nevertheless, we did not directly ask participants about their beliefs regarding the efficacy of the protective measures or the perceived barriers to their use. Therefore, additional information is needed to better understand the reasons for using or not using protective measures. One strength of our study is that both Barometer Health Surveys use identical methodologies, thus enabling yearly comparisons.

## Conclusion

Our study assessed the knowledge and practices regarding LB and tick bite prevention in France. A significant proportion of the French population has already been bitten by a tick and feels exposed to tick bites. Even though a low proportion of the population had never heard of LB, less than half of participants who were aware of LB considered themselves to be well informed about the risk and disease. We also showed that awareness and protective behaviors increased between 2016 and 2019 following the implementation of a national plan against tick-borne diseases in 2016. A better understanding of the barriers to using protective measures against tick bites is needed. It is therefore important that prevention campaigns focus on removing or reducing these barriers. Prevention campaigns should also focus on strengthening the recognition of *erythema migrans* to improve the early detection of the disease and prevent disseminated LB. Reducing infected tick densities and developing vaccines against tick bites or LB can only be seen as long-term solutions. Thus, strengthening information, increasing knowledge, and promoting preventive measures against tick bites are the main short-term interventions available to tackle tick-borne diseases. These measures are essential in order to decrease the LB incidence in France where it remains a frequent infection and can lead to rare but severe complications. This study will therefore help adapt and develop effective prevention interventions by taking into account the characteristics of target populations and increasing the knowledge and use of the recommended measures. These prevention programs for LB will also help prevent other tick-borne diseases.

## Data Availability

The datasets analysed during the current study are available from the corresponding author on reasonable request.

## Abbrevations

LB: Lyme Borreliosis.
aOR: Adjusted odds ratio.
95% CI: 95% Confidence interval.
